# Differential proteomics of lesional vs. non-lesional biopsies revealed non-immune mechanisms of alopecia areata

**DOI:** 10.1038/s41598-017-18282-1

**Published:** 2018-01-11

**Authors:** Kanchalit Thanomkitti, Rattiyaporn Kanlaya, Kedsarin Fong-ngern, Chompunoot Kapincharanon, Kanyarat Sueksakit, Prangwalai Chanchaem, Rattapon Thuangtong, Visith Thongboonkerd

**Affiliations:** 1 Department of Dermatology, Faculty of Medicine Siriraj Hospital, Mahidol University, Bangkok, Thailand; 2 Medical Proteomics Unit, Office for Research and Development, Faculty of Medicine Siriraj Hospital, Mahidol University, Bangkok, Thailand; 3 Center for Research in Complex Systems Science, Mahidol University, Bangkok, Thailand

## Abstract

Alopecia areata (AA) is one of the common hair disorders for which treatment is frequently ineffective and associated with relapsing episodes. Better understanding of disease mechanisms and novel therapeutic targets are thus required. From 10 AA patients, quantitative proteomics using LTQ-Orbitrap-XL mass spectrometer revealed 104 down-regulated, 4 absent, 3 up-regulated and 11 newly present proteins in lesional vs. non-lesional biopsies. Among these, the decreased levels of α-tubulin, vimentin, heat shock protein 70 (HSP70), HSP90, annexin A2 and α-enolase were successfully confirmed by Western blotting. Protein-protein interactions network analysis using STRING tool revealed that the most frequent biological processes/networks of the down-regulated proteins included tissue development, cell differentiation, response to wounding and catabolic process, whereas those for the up-regulated proteins included biological process, metabolic process, cellular transport, cellular component organization and response to stimulus. Interestingly, only 5 increased/newly present proteins were associated with the regulation of immune system, which may not be the predominant pathway in AA pathogenic mechanisms as previously assumed. In summary, we report herein the first proteome dataset of AA demonstrating a number of novel pathways, which can be linked to the disease mechanisms and may lead to discovery of new therapeutic targets for AA.

## Introduction

Alopecia areata (AA) is one of the common hair disorders characterized by sudden, non-scarring hair loss with a prevalence of approximately 0.2% of general population^1^. Although scalp is the most frequently affected part, AA can occur on other hair-bearing areas, e.g., eyebrow, eyelash, axilla, and body. Additionally, there are various spectra of the disease, including patchy AA, alopecia totalis, and alopecia universalis^2,3^. Moreover, nail involvement is not uncommon and observed in approximately 7–66% of AA patients. In some cases, AA may be associated with other autoimmune disorders, particularly autoimmune thyroiditis^4,5^. Nevertheless, the precise etiology and pathogenic mechanisms of AA remain unclear and deserve further elucidations. Moreover, treatment of AA is frequently ineffective and associated with relapsing episodes. This unfavorable outcome most likely reflects poor understanding of the disease pathogenesis and pathophysiology. Better understanding of disease mechanisms and novel therapeutic targets are thus required.

Proteomics has been emerged into biomedical research for more than two decades. It has been widely applied to several diseases with ultimate goals to better understand the disease mechanisms and to define novel biomarkers as well as new therapeutic targets^6,7^. Although it has been extensively applied to many other diseases, its applications to dermatology remain at an early phase. We thus employed proteomic tool to explore potentially novel mechanisms of AA by comparative analysis of proteins expressed in lesional vs. non-lesional biopsies (the self-controlled paired biopsies obtained from AA patients). The proteomic data were validated by Western blotting. Functional significance of the differential expression data relevant to AA disease mechanisms was then addressed by protein-protein interactions network analysis.

## Results

### Clinical characteristics of AA patients

Ten newly diagnosed patchy AA patients (7 males and 3 females; aged 41.2 ± 7.6 years) with single lesion (to ensure the homogeneity of the disease spectrum) were enrolled into this study. Among them, 20% had positive family history of AA. Severity of Alopecia Tool (SALT) scores were 5.6 ± 2.8. Histopathology confirmed the diagnosis of AA with reduced, but still detectable, hair follicles underneath without signs of fibrosis or chronicity. Additional demographic and clinical data are shown in Table 1.

**Table 1:** Demographic and clinical data of the AA patients.

Characteristics	Value
Number of participants	10
Age (years)	
Mean ± SD	41.2 ± 7.6
Min-max	29–55
Gender (%)	
Male	70
Female	30
Duration of hair loss (months) (mean ± SD)	1.5 ± 1.2
Family history of AA (%)	20
SALT score (mean ± SD)	5.6 ± 2.8

### Identification of differentially expressed proteins in lesional vs. non-lesional biopsies

**Figure 1:**
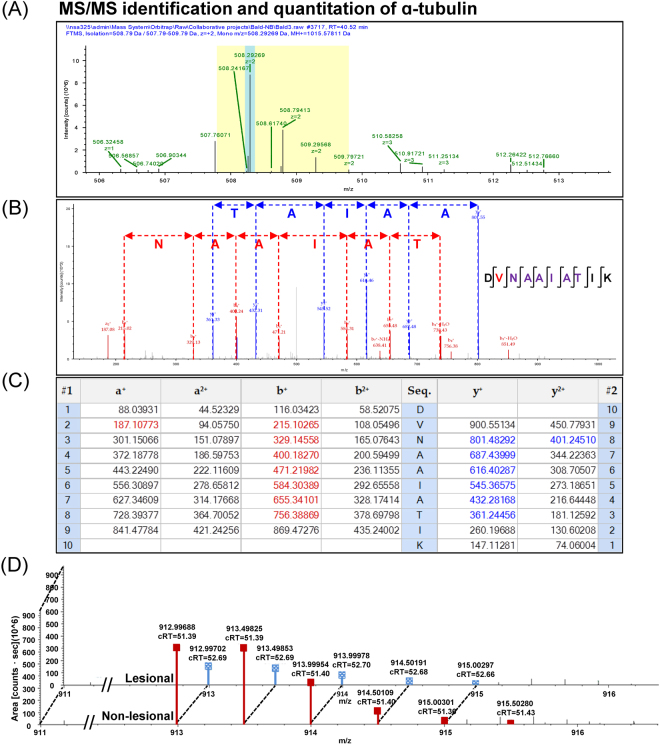
MS/MS identification and quantitative analysis of α-tubulin. (**A**) Isotope view of the selected MS precursor that was subjected to MS/MS peptide sequencing. (**B**) Graphical illustration of individual amino acid residues derived from MS/MS spectra that significantly matched to a peptide “DVNAAIATIK” of α-tubulin. (**C**) MS/MS ion table displaying the calculated mass of possible fragment ions used for such amino acid matching. In (**B**,**C**), fragment ions that were derived from N- and C-terminal scans of the spectra are highlighted in red and blue, respectively. (**D**) Representative MS/MS spectra of α-tubulin that were assigned for quantitative analysis to compare lesional vs. non-lesional biopsies.

**Figure 2:**
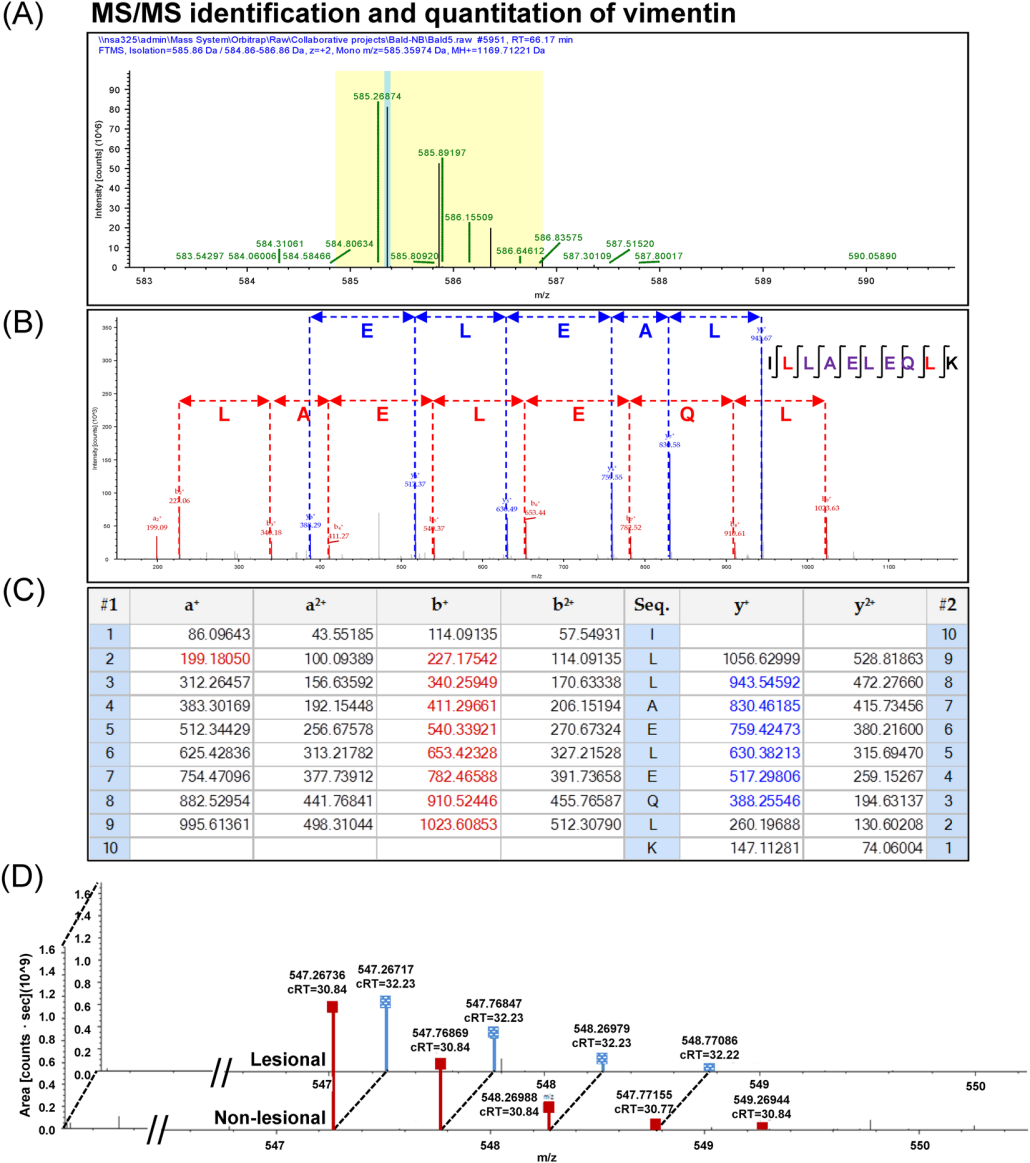
MS/MS identification and quantitative analysis of vimentin. (**A**) Isotope view of the selected MS precursor that was subjected to MS/MS peptide sequencing. (**B**) Graphical illustration of individual amino acid residues derived from MS/MS spectra that significantly matched to a peptide “ILLAELEQLK” of vimentin. **(C)** MS/MS ion table displaying the calculated mass of possible fragment ions used for such amino acid matching. In **(B**,**C)**, fragment ions that were derived from N- and C-terminal scans of the spectra are highlighted in red and blue, respectively. **(D)** Representative MS/MS spectra of vimentin that were assigned for quantitative analysis to compare lesional vs. non-lesional biopsies.

Using nanoLC-ESI-LTQ-Orbitrap tandem mass spectrometry (MS/MS) and high-stringent criteria for determination of significant differences by quantitative proteomics approach, a total of 122 proteins were identified as differentially expressed proteins in lesional vs. non-lesional biopsies. Their identities, identification scores, related mass spectral parameters and quantitative data are summarized in Table 2. Among these, 104 were decreased, 4 were absent, 3 were increased, and 11 were newly present in the lesional biopsies as compared to the non-lesional tissues (Table 2). Representative MS and MS/MS spectra of α-tubulin and vimentin, both of which were decreased in lesional biopsies, are shown in Figs 1A,B and 2A,B, respectively. In addition, matching of the MS/MS data with peptide sequences “DVNAAIATIK” of α-tubulin and “ILLAELEQLK” of vimentin are illustrated in Figs 1C and 2C, respectively. Moreover, quantitative analysis of peptides derived from α-tubulin and vimentin are demonstrated in Figs 1D and 2D, respectively.

**Table 2:** Summary of differentially expressed proteins in lesional vs. non-lesional biopsies of AA patients (in alphabetical order).

Accession no.	Protein name	MS/MS identification score	%Cov	MW (kDa)	p*I*	Abundance level (×10^9^ arbitrary unit) (Mean ± SEM)		Ratio(Lesional/Non-lesional)	*P-value*	
						Non-lesional	Lesional		*T-test*	*Mann-Whitney U test*
***Proteins whose levels were significantly decreased in lesional biopsies***										
P62258	14-3-3 protein epsilon	228	20.8	29.2	4.74	1.2665 ± 0.0661	0.4383 ± 0.1057	0.35	0.0030	0.0495
P31947	14-3-3 protein sigma	567	35.9	27.8	4.74	1.7498 ± 0.1048	0.5572 ± 0.0573	0.32	0.0007	0.0495
P63104	14-3-3 protein zeta/delta	412	26.5	27.7	4.79	1.5236 ± 0.1189	0.4528 ± 0.0725	0.30	0.0019	0.0495
P42765	3-ketoacyl-CoA thiolase, mitochondrial	106	6.6	41.9	8.09	0.0990 ± 0.0371	0.0096 ± 0.0096	0.10	0.0415	0.0463
P46782	40 S ribosomal protein S5	216	7.4	22.9	9.72	0.3306 ± 0.0547	0.1765 ± 0.0263	0.53	0.0357	0.0495
P11021	78 kDa glucose-regulated protein	126	3.4	72.3	5.16	0.5653 ± 0.0847	0.2668 ± 0.0176	0.47	0.0450	0.0495
P62736	Actin, aortic smooth muscle	4,301	62.3	42.0	5.39	11.5785 ± 0.7283	6.7962 ± 0.3618	0.59	0.0059	0.0495
P60709	Actin, cytoplasmic 1	6,695	62.7	41.7	5.48	15.3669 ± 0.9924	8.6725 ± 0.5307	0.56	0.0058	0.0495
P01009	Alpha-1-antitrypsin	673	28.7	46.7	5.59	0.6696 ± 0.0278	0.4381 ± 0.0299	0.65	0.0050	0.0495
P12814	Alpha-actinin-1	373	10.3	103.0	5.41	0.5002 ± 0.0507	0.1483 ± 0.0055	0.30	0.0033	0.0495
O43707	Alpha-actinin-4	372	7.6	104.8	5.44	0.4913 ± 0.0190	0.1666 ± 0.0234	0.34	0.0005	0.0495
P06733	Alpha-enolase	959	27.2	47.1	7.39	0.9960 ± 0.0307	0.5806 ± 0.0419	0.58	0.0016	0.0495
P04083	Annexin A1	998	28.3	38.7	7.02	0.6655 ± 0.0389	0.3032 ± 0.0180	0.46	0.0014	0.0495
P07355	Annexin A2	1,593	61.4	38.6	7.75	3.5947 ± 0.1765	1.8078 ± 0.1029	0.50	0.0012	0.0495
P06576	ATP synthase subunit beta, mitochondrial	721	18.7	56.5	5.40	0.7964 ± 0.0878	0.3916 ± 0.0360	0.49	0.0179	0.0495
P21810	Biglycan	479	29.9	41.6	7.52	1.2536 ± 0.1824	0.4363 ± 0.0325	0.35	0.0195	0.0495
P27482	Calmodulin-like protein 3	136	26.9	16.9	4.42	0.3238 ± 0.0557	0.0472 ± 0.0028	0.15	0.0125	0.0495
Q9NZT1	Calmodulin-like protein 5	341	41.8	15.9	4.44	0.6935 ± 0.0238	0.2509 ± 0.0180	0.36	0.0001	0.0495
P21926	CD9 antigen	128	4.4	25.4	7.15	0.6167 ± 0.0137	0.3275 ± 0.0185	0.53	0.0002	0.0495
P23528	Cofilin-1	284	40.4	18.5	8.09	0.6586 ± 0.1013	0.2740 ± 0.0170	0.42	0.0260	0.0495
P12109	Collagen alpha-1(VI) chain	1,779	21.4	108.5	5.43	4.0902 ± 0.3800	2.1863 ± 0.1420	0.53	0.0095	0.0495
Q05707	Collagen alpha-1(XIV) chain	251	3.7	193.4	5.30	0.1813 ± 0.0137	0.1194 ± 0.0073	0.66	0.0187	0.0495
P12110	Collagen alpha-2(VI) chain	1,013	18.1	108.5	6.21	2.0476 ± 0.1619	1.0137 ± 0.0788	0.50	0.0067	0.0495
P12111	Collagen alpha-3(VI) chain	6,673	26.4	343.5	6.68	3.3795 ± 0.0826	2.0208 ± 0.1211	0.60	0.0007	0.0495
P07585	Decorin	1,111	30.6	39.7	8.54	6.3779 ± 0.6144	2.3891 ± 0.1519	0.37	0.0047	0.0495
P17661	Desmin	849	32.6	53.5	5.27	1.8656 ± 0.0907	1.2064 ± 0.1184	0.65	0.0159	0.0495
P68104	Elongation factor 1-alpha 1	586	32.0	50.1	9.01	1.2573 ± 0.0907	0.3895 ± 0.0202	0.31	0.0010	0.0495
P49327	Fatty acid synthase	1,684	14.1	273.3	6.44	0.8729 ± 0.0160	0.6507 ± 0.0128	0.75	0.0004	0.0495
Q01469	Fatty acid-binding protein, epidermal	337	28.9	15.2	7.01	1.3691 ± 0.1281	0.2148 ± 0.0354	0.16	0.0011	0.0495
P04075	Fructose-bisphosphate aldolase A	220	19.0	39.4	8.09	0.7244 ± 0.0598	0.3043 ± 0.0135	0.42	0.0028	0.0495
P09382	Galectin-1	449	34.8	14.7	5.50	0.9392 ± 0.0723	0.4403 ± 0.0415	0.47	0.0057	0.0495
P47929	Galectin-7	2,026	72.1	15.1	7.62	4.7958 ± 0.6101	1.9441 ± 0.1443	0.41	0.0140	0.0495
P06396	Gelsolin	210	5.1	85.6	6.28	0.5783 ± 0.0399	0.3287 ± 0.0117	0.57	0.0027	0.0495
P09211	Glutathione S-transferase P	308	37.1	23.3	5.64	0.6136 ± 0.074	0.1990 ± 0.0115	0.32	0.0057	0.0495
P04406	Glyceraldehyde-3-phosphate dehydrogenase	1,618	43.6	36.0	8.46	2.3094 ± 0.1831	0.8372 ± 0.1037	0.36	0.0024	0.0495
P08107	Heat shock 70 kDa protein 1 A/1B	1,011	17.5	70.0	5.66	0.8604 ± 0.0111	0.3926 ± 0.0168	0.46	<0.0001	0.0495
P11142	Heat shock cognate 71 kDa protein	329	7.9	70.9	5.52	0.7670 ± 0.0840	0.3553 ± 0.0278	0.46	0.0133	0.0495
P04792	Heat shock protein beta-1	840	52.7	22.8	6.40	2.8791 ± 0.0417	0.8313 ± 0.0614	0.29	<0.0001	0.0495
P08238	Heat shock protein HSP 90-beta	115	6.2	83.2	5.03	0.5714 ± 0.0227	0.2134 ± 0.0136	0.37	0.0002	0.0495
P68871	Hemoglobin subunit beta	3,167	77.6	16.0	7.28	17.2633 ± 1.3237	8.7187 ± 0.3325	0.51	0.0049	0.0495
P02042	Hemoglobin subunit delta	1,383	47.6	16.0	8.05	8.2333 ± 0.3408	4.8357 ± 0.2541	0.59	0.0017	0.0495
P61978	Heterogeneous nuclear ribonucleoprotein K	117	2.6	50.9	5.54	0.3317 ± 0.0346	0.0924 ± 0.0463	0.28	0.0163	0.0495
P04908	Histone H2A type 1-B/E	878	35.4	14.1	11.05	4.6903 ± 0.2704	2.1839 ± 0.1125	0.47	0.0014	0.0495
Q96KK5	Histone H2A type 1-H	953	35.9	13.9	10.89	4.9275 ± 0.2597	2.2308 ± 0.1772	0.45	0.0013	0.0495
P62805	Histone H4	1,242	51.5	11.4	11.36	10.675 ± 0.4228	5.8368 ± 0.3738	0.55	0.0010	0.0495
P01859	Ig gamma-2 chain C region	755	23.3	35.9	7.59	3.6664 ± 0.2650	2.3450 ± 0.1638	0.64	0.0206	0.0495
P01860	Ig gamma-3 chain C region	525	19.1	41.3	7.90	4.5720 ± 0.3578	2.8894 ± 0.1940	0.63	0.0235	0.0495
P01834	Ig kappa chain C region	773	65.1	11.6	5.87	2.6707 ± 0.1593	1.6977 ± 0.1639	0.64	0.0185	0.0495
P14923	Junction plakoglobin	514	22.6	81.7	6.14	0.9346 ± 0.0735	0.3792 ± 0.0671	0.41	0.0046	0.0495
P13645	Keratin, type I cytoskeletal 10	6,621	50.5	58.8	5.21	16.6131 ± 1.6321	9.1721 ± 0.7156	0.55	0.0226	0.0495
P02533	Keratin, type I cytoskeletal 14	7,774	72.3	51.5	5.16	16.6131 ± 1.6321	9.1721 ± 0.7156	0.55	0.0226	0.0495
P19012	Keratin, type I cytoskeletal 15	2,200	38.4	49.2	4.77	14.0326 ± 1.0307	7.7638 ± 0.6192	0.55	0.0092	0.0495
P08779	Keratin, type I cytoskeletal 16	4,060	57.5	51.2	5.05	16.6131 ± 1.6321	9.1721 ± 0.7156	0.55	0.0226	0.0495
Q04695	Keratin, type I cytoskeletal 17	3,578	62.7	48.1	5.02	14.0326 ± 1.0307	7.7638 ± 0.6192	0.55	0.0092	0.0495
P78385	Keratin, type II cuticular Hb3	1,083	28.8	54.2	5.64	2.0191 ± 0.1673	0.2130 ± 0.1065	0.11	0.0010	0.0495
P78386	Keratin, type II cuticular Hb5	1,096	28.8	55.8	6.55	1.6105 ± 0.1287	0.2130 ± 0.1065	0.13	0.0014	0.0495
O43790	Keratin, type II cuticular Hb6	1,239	37.7	53.5	5.66	2.1958 ± 0.1612	0.2130 ± 0.1065	0.10	0.0006	0.0495
Q7Z794	Keratin, type II cytoskeletal 1b	584	8.7	61.9	5.99	13.5626 ± 1.8751	5.3357 ± 0.3783	0.39	0.0177	0.0495
P35908	Keratin, type II cytoskeletal 2 epidermal	2,686	37.7	65.4	8.00	18.4882 ± 0.9864	8.3626 ± 0.2635	0.45	0.0006	0.0495
Q01546	Keratin, type II cytoskeletal 2 oral	1,657	10.7	65.8	8.12	10.6049 ± 0.3755	4.3335 ± 0.3619	0.41	0.0003	0.0495
P13647	Keratin, type II cytoskeletal 5	7,660	55.6	62.3	7.74	24.0143 ± 1.1424	8.3270 ± 0.2756	0.35	0.0002	0.0495
P02538	Keratin, type II cytoskeletal 6 A	6,029	54.1	60.0	8.00	24.0143 ± 1.1424	8.3270 ± 0.2757	0.35	0.0002	0.0495
P04259	Keratin, type II cytoskeletal 6B	5,349	53.7	60.0	8.00	24.0143 ± 1.1424	8.3270 ± 0.2758	0.35	0.0002	0.0495
P48668	Keratin, type II cytoskeletal 6 C	5,772	54.1	60.0	8.00	24.0143 ± 1.1424	8.3270 ± 0.2759	0.35	0.0002	0.0495
P08729	Keratin, type II cytoskeletal 7	762	16.0	51.4	5.48	8.8710 ± 0.2204	3.5040 ± 0.2806	0.39	0.0001	0.0495
Q3SY84	Keratin, type II cytoskeletal 71	263	5.5	57.3	6.61	10.2813 ± 1.1519	4.2346 ± 0.3536	0.41	0.0058	0.0495
O95678	Keratin, type II cytoskeletal 75	2,651	16.0	59.5	7.74	16.602 ± 0.9683	6.6123 ± 0.2142	0.40	0.0006	0.0495
Q5XKE5	Keratin, type II cytoskeletal 79	2,074	29.4	57.8	7.20	17.0162 ± 0.8674	6.6551 ± 0.1142	0.39	0.0003	0.0495
P05787	Keratin, type II cytoskeletal 8	316	11.4	53.7	5.59	10.6607 ± 1.4632	2.9471 ± 0.2498	0.28	0.0055	0.0495
P00338	L-lactate dehydrogenase A chain	201	12.7	36.7	8.27	0.5838 ± 0.0201	0.3102 ± 0.0350	0.53	0.0029	0.0495
P33121	Long-chain-fatty-acid–CoA ligase 1	44	5.0	77.9	7.15	0.4860 ± 0.1132	0.0508 ± 0.0508	0.10	0.0340	0.0495
P51884	Lumican	1,839	41.7	38.4	6.61	4.5287 ± 0.2844	2.0393 ± 0.1228	0.45	0.0018	0.0495
P14174	Macrophage migration inhibitory factor	203	7.8	12.5	7.88	1.5961 ± 0.0858	0.6650 ± 0.0276	0.42	0.0004	0.0495
P20774	Mimecan	1,453	36.6	33.9	5.63	2.1421 ± 0.1143	0.9376 ± 0.0647	0.44	0.0008	0.0495
Q9Y5U8	Mitochondrial pyruvate carrier 1	36	6.4	12.3	9.61	0.9031 ± 0.0395	0.4261 ± 0.0353	0.47	0.0010	0.0495
P60660	Myosin light polypeptide 6	502	42.4	16.9	4.65	0.8961 ± 0.0351	0.5230 ± 0.0436	0.58	0.0033	0.0495
P35749	Myosin-11	1,408	11.6	227.2	5.50	0.9947 ± 0.0843	0.5709 ± 0.0724	0.57	0.0293	0.0495
P35579	Myosin-9	939	6.7	226.4	5.60	0.8154 ± 0.0461	0.4245 ± 0.0208	0.52	0.0021	0.0495
Q09666	Neuroblast differentiation-associated protein AHNAK	85	6.0	628.7	6.15	0.5197 ± 0.0773	0.2652 ± 0.0175	0.51	0.0268	0.0495
P62937	Peptidyl-prolyl cis-trans isomerase A	1,176	54.6	18.0	7.81	1.2188 ± 0.1121	0.7801 ± 0.0933	0.64	0.0487	0.0495
Q06830	Peroxiredoxin-1	382	29.7	22.1	8.13	0.8360 ± 0.0346	0.5654 ± 0.0666	0.68	0.0176	0.0495
P00558	Phosphoglycerate kinase 1	136	10.6	44.6	8.10	0.6724 ± 0.1006	0.2890 ± 0.0233	0.43	0.0361	0.0495
Q13835	Plakophilin-1	335	12.1	82.8	9.13	0.3616 ± 0.0808	0.0941 ± 0.0472	0.26	0.0417	0.0495
Q15149	Plectin	217	1.0	531.5	5.96	0.2542 ± 0.0378	0.0966 ± 0.0130	0.38	0.0266	0.0495
P0CG48	Polyubiquitin-C	331	60.4	77.0	7.66	1.1121 ± 0.0596	0.4721 ± 0.0251	0.42	0.0006	0.0495
P02545	Prelamin-A/C	1,107	30.1	74.1	7.02	1.5438 ± 0.0733	0.8505 ± 0.0645	0.55	0.0027	0.0495
P07737	Profilin-1	293	32.1	15.0	8.27	0.6371 ± 0.0404	0.1381 ± 0.0770	0.22	0.0056	0.0495
P51888	Prolargin	1,056	28.8	43.8	9.38	2.4598 ± 0.0821	0.9291 ± 0.0330	0.38	0.0001	0.0495
P31151	Protein S100-A7	158	21.8	11.5	6.77	0.4665 ± 0.0147	0.1472 ± 0.0740	0.32	0.0148	0.0495
P05109	Protein S100-A8	447	44.1	10.8	7.03	1.2158 ± 0.0614	0.4230 ± 0.0946	0.35	0.0026	0.0495
P06702	Protein S100-A9	197	30.7	13.2	6.13	0.7494 ± 0.0541	0.0672 ± 0.0672	0.09	0.0017	0.0463
Q92928	Putative Ras-related protein Rab-1C	328	19.4	22.0	5.43	0.2738 ± 0.0119	0.1079 ± 0.0551	0.39	0.0488	0.0495
P14618	Pyruvate kinase PKM	520	27.1	57.9	7.84	0.5852 ± 0.0649	0.2305 ± 0.0392	0.39	0.0094	0.0495
P50395	Rab GDP dissociation inhibitor beta	455	6.7	50.6	6.47	0.0834 ± 0.0178	0.0103 ± 0.0130	0.16	0.0223	0.0463
P02787	Serotransferrin	1,163	27.2	77.0	7.12	0.7634 ± 0.0400	0.5552 ± 0.0292	0.73	0.0214	0.0495
P02768	Serum albumin	10,830	73.6	69.3	6.28	28.8146 ± 0.6575	18.7806 ± 0.6334	0.65	0.0004	0.0495
Q01995	Transgelin	362	29.4	22.6	8.84	0.6888 ± 0.0147	0.4063 ± 0.0265	0.59	0.0008	0.0495
Q07283	Trichohyalin	75	1.2	253.8	5.78	0.4259 ± 0.0398	0.0227 ± 0.0227	0.05	0.0008	0.0463
P68363	Tubulin alpha-1B chain	865	29.1	50.1	5.06	1.5395 ± 0.0642	0.5023 ± 0.0184	0.33	0.0001	0.0495
P68366	Tubulin alpha-4A chain	297	19.6	49.9	5.06	1.5094 ± 0.0899	0.4145 ± 0.0407	0.27	0.0004	0.0495
Q13509	Tubulin beta-3 chain	789	22.0	50.4	4.93	1.8837 ± 0.3747	0.5554 ± 0.0613	0.29	0.0334	0.0495
Q9H943	Uncharacterized protein C10orf68	33	1.1	71.5	9.35	1.032 ± 0.1089	0.4478 ± 0.0440	0.43	0.0119	0.0495
P08670	Vimentin	3,962	50.2	53.6	5.12	4.2836 ± 0.1597	2.5214 ± 0.1212	0.59	0.0008	0.0495
P02774	Vitamin D-binding protein	120	5.5	52.9	5.54	0.0895 ± 0.0012	0.0040 ± 0.0040	0.05	<0.0001	0.0463
***Proteins that were absent in lesional biopsies***										
P00918	Carbonic anhydrase 2	132	6.2	29.2	7.40	0.0193 ± 0.0117	0.0000 ± 0.0000	0.00	<0.0001	0.1213
O76009	Keratin, type I cuticular Ha3-I	1,046	45.5	45.9	4.82	1.8303 ± 0.1286	0.0000 ± 0.0000	0.00	0.0001	0.0369
P36952	Serpin B5	27	2.4	42.1	6.05	0.3940 ± 0.0095	0.0000 ± 0.0000	0.00	<0.0001	0.0369
P40939	Trifunctional enzyme subunit alpha, mitochondrial	124	3.8	82.9	9.04	0.0726 ± 0.0231	0.0000 ± 0.0000	0.00	0.0190	0.0369
***Proteins whose levels were significantly increased in lesional biopsies***										
Q15323	Keratin, type I cuticular Ha1	1,693	48.8	47.2	4.88	6.0527 ± 0.1399	8.9227 ± 0.6585	1.47	0.0119	0.0495
Q14525	Keratin, type I cuticular Ha3-II	1,769	37.9	46.2	4.84	6.0527 ± 0.1399	8.9227 ± 0.6585	1.47	0.0119	0.0495
P52746	Zinc finger protein 142	83	0.4	187.8	7.91	1.7416 ± 0.1625	4.0107 ± 0.3818	2.30	0.0048	0.0495
***Proteins that were newly present in lesional biopsies***										
Q07020	60 S ribosomal protein L18	121	6.9	21.6	11.72	0.0000 ± 0.0000	0.1001 ± 0.0008	#DIV/0!	<0.0001	0.0369
P19652	Alpha-1-acid glycoprotein 2	252	8.5	23.6	5.11	0.0000 ± 0.0000	0.0910 ± 0.0141	#DIV/0!	0.0029	0.0369
P08311	Cathepsin G	62	6.7	28.8	11.19	0.0000 ± 0.0000	0.3751 ± 0.0527	#DIV/0!	0.0021	0.0369
P02671	Fibrinogen alpha chain	129	3.1	94.9	6.01	0.0000 ± 0.0000	0.2659 ± 0.0124	#DIV/0!	<0.0001	0.0369
P02679	Fibrinogen gamma chain	85	17.4	51.5	5.62	0.0000 ± 0.0000	0.3585 ± 0.0171	#DIV/0!	<0.0001	0.0369
P02790	Hemopexin	62	9.7	51.6	7.02	0.0000 ± 0.0000	0.3937 ± 0.0171	#DIV/0!	<0.0001	0.0369
P30086	Phosphatidylethanolamine-binding protein 1	183	35.3	21.0	7.53	0.0000 ± 0.0000	0.2233 ± 0.0418	#DIV/0!	0.0059	0.0369
P30101	Protein disulfide-isomerase A3	49	2.2	56.7	6.35	0.0000 ± 0.0000	0.1212 ± 0.0039	#DIV/0!	<0.0001	0.0369
P26447	Protein S100-A4	29	7.9	11.7	6.11	0.0000 ± 0.0000	0.4159 ± 0.0455	#DIV/0!	0.0008	0.0369
P38646	Stress-70 protein, mitochondrial	119	1.8	73.6	6.16	0.0000 ± 0.0000	0.0937 ± 0.0087	#DIV/0!	0.0004	0.0369
P12956	X-ray repair cross-complementing protein 6	52	3.1	69.8	6.64	0.0000 ± 0.0000	1.1869 ± 0.1049	#DIV/0!	0.0003	0.0369

%Cov = %Sequence coverage [(number of the matched residues/total number of residues in the entire sequence) × 100%].

^#^DIV/0! = Divided by zero (not present in non-lesional area, but newly present in lesional area).

### Confirmation of the proteomic data by Western blotting

**Figure 3:**
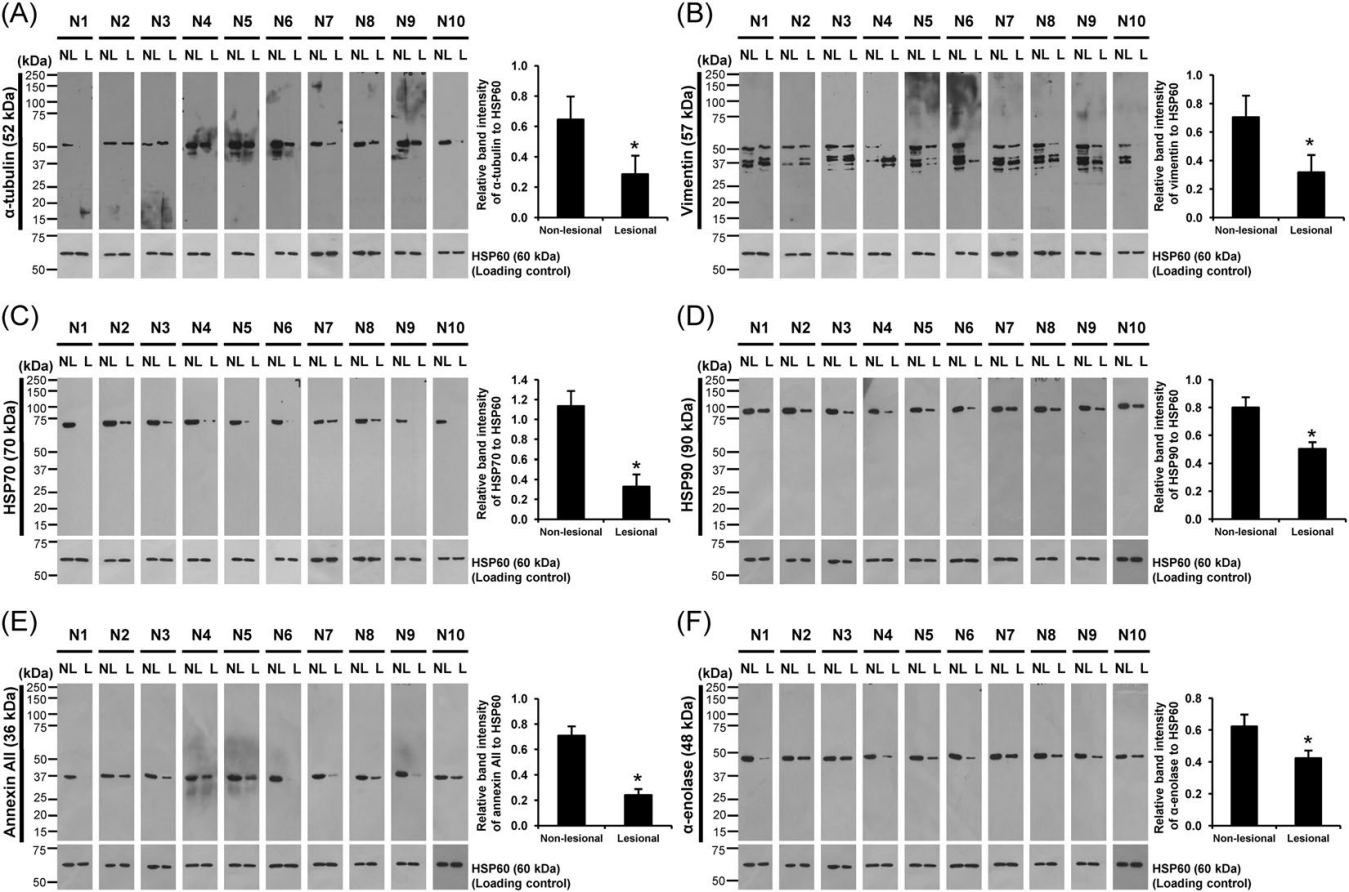
Validation of the proteomic data and quantitative analysis by Western blot analysis. Western blot analysis of protein lysates derived from lesional and non-lesional biopsies (30 µg/lane) obtained from ten individual patients using mouse monoclonal anti-α-tubulin **(A)**, anti-vimentin **(B)**, anti-HSP70 **(C)** or anti-HSP90 **(D)**, or goat polyclonal anti-annexin A2 **(E)**, or rabbit polyclonal anti-α-enolase antibody **(F)**, as the primary antibody. Note that HSP60 served as the loading control. Quantification data of band intensity of each protein was normalized with that of the loading control (HSP60) and are presented as mean ± SEM (n = 10 individual biopsies). *p < 0.05 vs. non-lesional biopsies.

Because proteins whose levels were significantly decreased in the lesional biopsies were predominate in the list, we confirmed the decreased levels of α-tubulin, vimentin, HSP70, HSP90, annexin A2 and α-enolase determined by quantitative proteomics by another method, i.e., Western blotting. Because quantitative proteomics revealed significant changes of proteins that are commonly used as loading controls, i.e., actin and glyceraldehyde-3-phosphate dehydrogenase (GAPDH) – see Table 2, whereas there were no significant changes in level of HSP60 observed (as such, this protein was not included in the list of significantly altered proteins shown in Table 2), HSP60 was selected to serve as the loading control to normalize levels of all the aforementioned proteins in our present study. The data showed that levels of α-tubulin, vimentin, HSP70, HSP90, annexin A2 and α-enolase normalized with HSP60 were significantly decreased in all 10 individual AA patients (Fig. 3), consistent with the quantitative proteomics data.

### Functional classification and global protein network analysis

**Figure 4:**
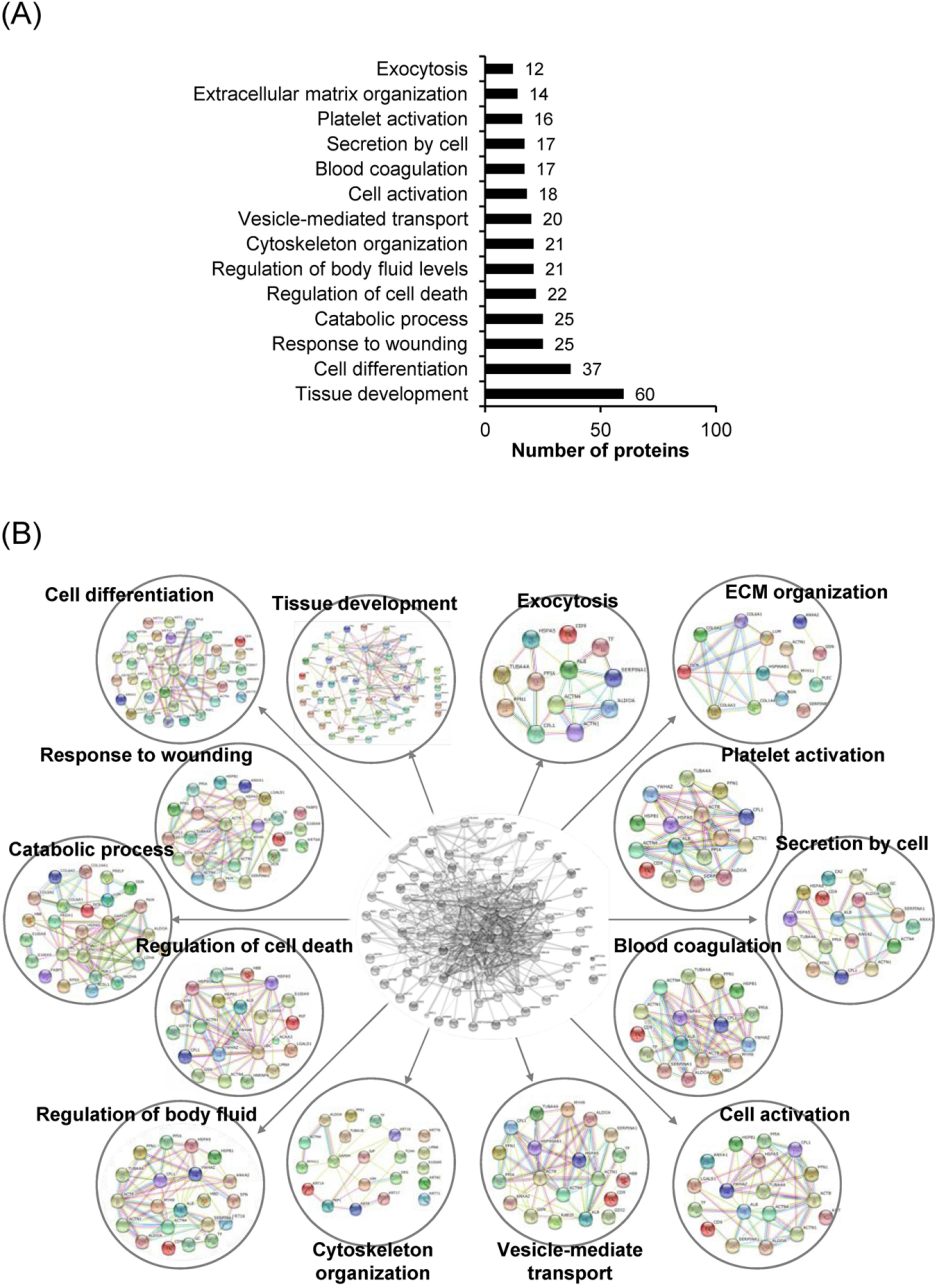
Functional classification and global protein network analysis of the down-regulated proteins. (**A**) Functional enrichment using gene ontology. **(B)** Protein-protein interactions network of the identified proteins that were significantly decreased or absent in the lesional biopsies as compared to the non-lesional samples.

**Figure 5:**
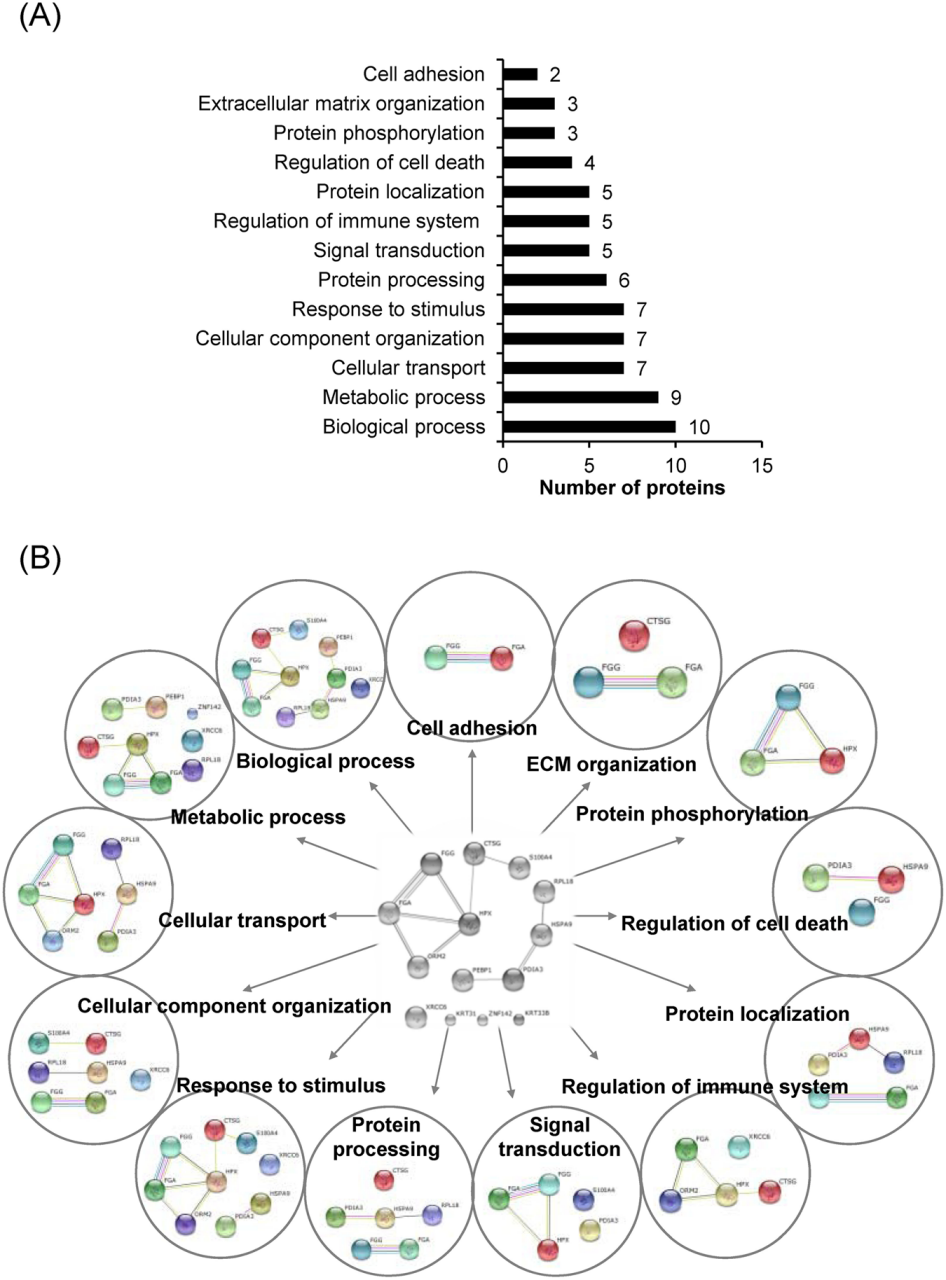
Functional classification and global protein network analysis of the up-regulated proteins. (**A**) Functional enrichment using gene ontology. **(B**) Protein-protein interactions network of the identified proteins that were significantly increased or newly present in the lesional biopsies as compared to the non-lesional samples.

The differentially expressed proteins were classified by STRING tool. From a total of 108 decreased/absent proteins in lesional biopsies, the top-three most frequent biological processes/networks included tissue development (60 proteins), cell differentiation (37 proteins), response to wounding (25 proteins), and catabolic process (25 proteins) (Fig. 4A and B). For the 14 increased/newly present proteins in lesional biopsies, the top-three most frequent biological processes/networks included biological process (10 proteins), metabolic process (9 proteins), cellular transport (7 proteins), cellular component organization (7 proteins), and response to stimulus (7 proteins) (Fig. 5A and B). Interestingly, only 5 increased/newly present proteins were associated with the regulation of immune system (Fig. 5A and B), which may not be the predominant pathway in AA pathogenic mechanisms as previously assumed.

## Discussion

AA, one of the common hair disorders, is characterized by oval or round, well-circumscribed balding patch(es). Some patients with limited area of AA may have spontaneous recovery and experience only a single AA episode in their lifetime. However, a much larger proportion of the patients have persistent AA that is resistant to medical therapy or have chronic relapsing episodes of the disease. Previous knowledge had suggested that the pathogenesis of AA is related to destruction of the hair follicles by immune process, particularly via cooperative roles of both CD8^+^ and CD4^+^ T lymphocytes^8,9^. In fact, the pathogenesis of AA and mechanisms of failure in hair follicle formation remain unclear and largely unknown. This study thus aimed to address such mechanisms and to explore previously unknown or hidden mechanisms associated with defective hair follicles and development of AA using a recent advanced technology based on quantitative proteomics followed by protein-protein interactions network analysis.

Quantitative proteomics revealed 122 differentially expressed proteins in lesional vs. non-lesional biopsies. From these, we performed Western blotting to confirm the differential expression data obtained from a quantitative proteomics approach. Vimentin and tubulin were selected because of their significant roles in hair follicular development. Vimentin is an intermediate filament cytoskeleton, which is also known to serve as a marker for mesenchymal feature that can be found among mesenchymal cell populations required for the development of hair follicles^10,11^. Tubulin is a main component of microtubule, a structure that also involves in cellular development and function, i.e., mitosis, vesicular trafficking, cell motility, and wound healing^12^. It is also plays a role in pigment transport^13,14^. In addition, we also performed Western blotting to confirm changes in levels of several other proteins, including HSP70, HSP90, annexin A2 and α-enolase. Using HSP60 as the loading control, the Western blot data nicely confirmed significant changes in levels of these proteins (Fig. 3), consistent with the data obtained from quantitative proteomics approach (Table 2).

Global protein network analysis was then performed to obtain functional insights of the identified proteins that were differentially expressed. From 14 increased/newly presented proteins, only five proteins were classified to get involved in immune-mediated mechanisms (Fig. 5A and B). Among these, fibrinogen alpha and gamma chains were found to be related to various biological functions including immune mechanism (Fig. 5B). Although previous studies have reported the potential roles of fibrinogen in wound healing, angiogenesis and inflammatory response in epithelial cells via both innate and T lymphocyte-mediated pathways^15,16^, the evidence of its association with AA and hair loss had never been reported. Interestingly, a recent study has revealed that fibrinogen could induce activation and recruitment of myelin-specific Th1 cells and peripheral macrophages into the central nervous system causing demyelination and autoimmune encephalomyelitis^17^. However, a precise role of fibrinogen as a driver of immune privilege breakdown of hair follicles observed in AA remains unclear and deserves further investigations.

Hemopexin (HPX) was found as one of the newly present proteins in lesional biopsies. HPX is a heme-binding glycoprotein that also serves as an antioxidant or oxidative stress scavenger. Previous studies have reported decreased plasma concentration of HPX in severe intravascular hemolysis, chronic hemolytic anemia, chronic liver disease, and acute porphyria attack^18^. In the context of autoimmune disease, HPX plays an important role in mercury-induced autoimmunity^19,20^. The data have shown that HPX-null mice had less number of B and activated T cells as well as lower autoantibody production. T cells isolated from mercury-treated HPX-null mice also had a reduction of IFN-γ production. These results suggest that HPX may involve in autoimmune diseases via regulating heme-iron homeostasis and IFN-γ response^19,20^. Additionally, there is a report of the increased HPX in vernal keratoconjuctivitis, a chronic allergic inflammatory disease^21^. However, the role of HPX in hair disorder had not been studied previously. The presence of HPX only in lesional area of AA might suggest its role in inflammatory process leading to chronicity in AA. Nevertheless, the precise role of HPX in AA needs further elucidations.

Another protein that was exclusively expressed in lesional biopsies was α1-acid glycoprotein 2 (also known as orosomucoid 2). This protein has immunomodulatory effect and can inhibit mitogenic response of lymphocytes, in particular, T cell population^22^. On the other hand, orosomucoid 2 can stimulate T cell proliferation at low concentration and induce mononuclear cells to produce several cytokines involving inflammatory response^23,24^. Additionally, it is known that glycosylation patterns of orosomucoid 2 are distinct among inflammatory and autoimmune diseases (e.g., rheumatoid arthritis, SLE, autoimmune thyroiditis, etc.), thereby, affecting their physical properties and function^25–27^. Our findings may suggest the role of this protein in stimulation of proliferating T cells and regulation of local inflammation in lesional area. Knockdown of orosomucoid 2 or investigations on its glycan moieties during the disease onset may be useful to gain mechanistic insights of its increase in AA.

In addition to fibrinogen, HPX and orosomucoid 2, x-ray repair complementing defective repair in chinese hamster cells 6 (XRCC6) was also listed as the differentially expressed proteins involving in immune-mediated mechanism. XRCC6 (also known as Ku70) is a single-strand DNA-dependent/ATP-dependent helicase that plays role in chromosome translocation and double-strand break DNA repair. Interestingly, it has been reported that individuals with SLE produced reactive autoantibody to XRCC6^28^. Additionally, polymorphisms of XRCC6 are associated with the risk of SLE susceptibility^29^. In the context of AA, the decrease in XRCC4 mRNA has been previously reported although its precise mechanism remains unknown^30^.

Surprisingly, functional classification and protein network prediction did not show immune-mediated mechanism as the predominant pathway involved in AA as we initially anticipated. This surprising result indicated that there should be several other non-immune mechanisms that are involved in the pathogenic mechanisms of AA that might be previously unknown, unexplored and/or hidden by limited knowledge in the past. Using recent advanced proteomic technology helped us to explore previously unknown, unexplored and/or hidden mechanisms/pathways in an unbiased manner as in the case of many other diseases^6,7,31–35^. Interestingly, a much larger number of the differentially expressed proteins (approximately 89%) had decreased levels or were absent in the AA lesional biopsies. The most frequent biological processes/networks of these down-regulated proteins included tissue development, cell differentiation, response to wounding and catabolic process (Fig. 4).

In concordance to the previous transcriptomics studies of AA^30,36,37^, we found the decreased expression of both Type I and Type II keratin in the lesional biopsies (including 20 keratin species as follows: K2, K5-K8, K14-17, K71, K75-77, K79, K83, and K85-86). These proteins are essential in hair and nail formation. Among these, K75 and K86 have been previously reported to be increased in response to corticosteroid treatment in AA patients, suggesting that its increase may be used as a biomarker for monitoring response to the steroid therapy^38^. In addition, several genes in S100 family were decreased in AA patients as compared to healthy controls^38^. We also found the decreased levels of S100A7, S100A8, and S100A9 in lesional biopsies. S100A8, S100A9 and S100A8/A9 heterodimers have been reported to play roles in neutrophil chemotaxis and adhesion to inflammatory sites^39,40^. They are also known as myeloid-related proteins that are highly expressed in neutrophils, monocytes, differentiated macrophages and keratinocytes^41^. It is thus plausible that the decreased levels of S100A8 and S100A9 found in AA tissues might reflect restriction of the immune response in the affected area as well as dysregulation of keratinocyte proliferation and differentiation^42^.

In addition, we compared our data to the previously reported differential proteomes of hair follicles at different phases in mice^43^. In concordance to the previous findings, we observed the decreased expression of annexin A1, heat shock protein (HSP)-β1 and vimentin, which were also decreased in telogen-hair follicle, but increased in anagen and catagen phases^43^. These provide further support to the observation that hair follicles in patients with AA rapidly progress from anagen to telogen phase, re-enter to anagen phase, and then strictly reside in anagen III/IV phase. Taken together, the decreased levels of these proteins support the miniature of hair follicles found in AA patients. Moreover, we also compared our proteomic data to the genomic data obtained from genome-wide association study (GWAS) and linkage analysis in AA patients^30,44–48^. In these studies, several immune-related genes have been identified so far, while only a few of non-immune genes have been reported^49,50^. These genes include *ERBB3, VDR, STX17*, *PRDX5*, *KIAA0350/CLEC16A* and *SPATA5*. Among these, *ERBB3, VDR, STX17* and *PRDX5* are skin/hair-related genes^51,52^. Herein, we provide additional dataset of non-immune proteins mainly involved in tissue development and differentiation. Although our data is different from the previous GWAS reports, these discrepancies could be explained by differences in technical approach. Another important factor is the difference in populations of AA patients included among these studies. Since several lines of evidence have suggested that AA is strongly related to genetic basis; therefore, different ethnic populations should be taken into account for such differences.

Besides the genetics, it should be noted that tissue biopsies with different onsets and severities among the studies could also affect the results at transcriptome and proteome levels. Moreover, our present study showed decreased levels of several cytoskeletal/structural proteins in the lesional areas. It was thus possible that their decreases might be a result from the reduction of hair follicles in the lesional areas. Alternatively, these altered proteome may serve as the potentially novel mechanisms leading to AA. Nevertheless, further functional investigations on these candidate biological functions/pathways should be done to strengthen our hypothesis.

In summary, we report herein the first proteome dataset of AA, which implicates that a number of potentially novel mechanisms or biological pathways may be involved in pathogenic mechanisms of AA. Our data offer opportunities to explore previously unknown, unexplored or hidden mechanisms of AA and to define novel biomarkers for diagnostics/prognostics and new therapeutic targets for better clinical outcome for AA.

## Materials and Methods

### Patients and lesional/non-lesional biopsies

This study was approved by the institutional ethical committee (Siriraj Institutional Review Board) (approval no. Si259/2015). All the experiments involved human subjects and clinical samples were conducted according to the international guidelines, i.e. the Declaration of Helsinki, the Belmont Report, and ICH Good Clinical Practice, and informed consents were obtained from all subjects. Newly diagnosed patchy AA patients were recruited during June – October 2015 and subjected to skin biopsy of both lesional and non-lesional areas. Patients with any of the following exclusion criteria, including psychiatric disorder (e.g., trichotillomania), active scalp infection, systemic conditions affecting the scalp and hairs (e.g., anemia), recurrent AA, history of vitamin and/or mineral supplement, history of coagulopathy, ingestion of anti-platelets and/or anticoagulants, pregnancy and lactation, were excluded. Clinical data from each patient were recorded, including age, gender, duration from disease onset to the visit, family history of AA, location, underlying disease, and Severity of Alopecia Tool Score (SALT score). Finally, ten enrolled patients were included in this study. Thereafter, four punch biopsies were obtained: the first two were from lesional area for histopathological examination to confirm the diagnosis of AA; the third was from the lesional area for proteome analysis; and the fourth was from non-lesional area (defined as the scalp region that was away from the margin of the lesional area more than a diameter of the lesion) for proteome analysis.

### Protein extraction

Biopsies were taken from both non-lesional and lesional areas of each patient and proteins were extracted from each biopsy separately. Briefly, the biopsied tissue was chopped into small pieces and washed with pre-chilled phosphate buffered saline (PBS). The sample was snap frozen by liquid nitrogen, ground into powder, extracted by SDT lysis buffer (containing 4% SDS, 100 mM DTT, and 100 mM Tris-HCl; pH 7.6) and incubated on ice for 30 min. The supernatant was collected after centrifugation at 10,000 × *g* and 4 °C for 30 min and protein concentration was measured by Bio-Rad Protein Assay (Bio-Rad Laboratories; Hercules, CA) based on the Bradford’s method.

### In-solution tryptic digestion by filter-aided sample preparation (FASP) method

Equal amount of total protein derived from each sample was pooled and digested by trypsin according to FASP protocol^53^. Briefly, the protein mixture in SDT buffer was reduced by heating at 95 °C for 5 min. After cooling down at RT, the sample was transferred to an Omega Nanosep 10 K device (Pall Corporation; Port Washington, NY), added with 200 µl of 8 M urea in 100 mM Tris-HCl (pH 8.5), and then centrifuged at 14,000 × *g* and RT for 15 min. This buffer exchange step was repeated one more cycle. The recovered proteins were then alkylated with 100 µl of 50 mM iodoacetamide in 8 M urea/100 mM Tris-HCl (pH 8.5) at RT in the dark using a ThermoMixer^®^ C (Eppendorf; Hauppauge, NY) for 20 min. Thereafter, buffer exchange was performed twice by centrifugation at 14,000 × *g* and RT for 15 min each using 200 µl of 8 M urea/100 mM Tris-HCl (pH 8.5). The proteins were then finally exchanged into 50 m NH_4_HCO_3_ and then digested with sequencing grade modified trypsin (Promega; Madison, WI) in 50 mM NH_4_HCO_3_ at a ratio of 1:50 (w/w) trypsin/protein at 37 °C for 16 h in a ThermoMixer^®^ C. The digested peptides were collected by transferring the filter unit to a new collection tube and centrifuged at 14,000 × *g* at 25 °C for 15 min. Trypsin activity was then stopped by adding 10 µl of 5% formic acid in 80% acetronitrile (ACN), and the digested peptides were dried by a vacuum concentrator (ScanVac; Lynge, Denmark). The peptides were finally resuspended in 0.1% formic acid prior to tandem mass spectrometry (MS/MS).

### nanoLC-ESI-LTQ-Orbitrap MS/MS

Each sample was run in technical triplicates. Separation of the digested peptides was performed using EASY-nLC II (Thermo Scientific; Waltham, MA). Briefly, peptides were loaded from a cooled (7 °C) autosampler into an in-house, 3-cm-long pre-column containing 5-µm C18 resin (Dr.Maisch GmbH; Ammerbuch, Germany) and then to an in-house, 10-cm-long analytical column packed with 3-µm C18 resin (Dr. Maisch GmbH) using mobile phase A (0.1% formic acid). The peptides were then separated by mobile phase B (ACN/0.1% formic acid) gradient elution with four steps as follows: 2–9% for 15 min, 9–35% for 85 min, 35–95% for 20 min, and then 95% for 10 min at a flow rate of 200 nl/min. Peptide sequences were then analyzed by LTQ-Orbitrap-XL (Thermo Scientific) in positive mode with ESI nanosprayer ion source.

Data were acquired in a collision-induced dissociation (CID) top-12 mode under the control of the Xcalibur 2.1.0 and LTQ Tune Plus 2.5.5 software (Thermo Scientific). The cycle of one full scan was performed at a resolution of 30,000 (300–2,000 *m/z*) in the Orbitrap followed by 12 data-dependent MS/MS scans in the linear ion trap with enabled preview mode for FTMS master scan. The minimum signal threshold at 1 × 10^5^ was required for a precursor ion to be selected for further fragmentation. Accumulation target values of full MS and MS/MS scan were 5 × 10^5^ and 3 × 10^4^ ions, respectively. Singly charged ions and unassigned charge states were excluded for fragmentation. Helium was used as a collision gas and the normalized collision energy was set at 35%. The activation time was 30 ms for acquiring mass spectra. The duration of dynamic exclusion was 180 s.

### MS/MS spectral interpretation and quantitative analysis

The MS/MS raw spectra were deconvoluted and then extracted into output searchable*.mgf* files using Proteome Discoverer v.1.4.1.14 software (Thermo Scientific). Mascot software version 2.4.0 (Matrix Science; London, UK) was used to search MS/MS spectra against SwissProt database of mammalian with the following standard Mascot parameters for CID: Enzyme = trypsin, maximal number of missed cleavages = 1, peptide tolerance = ±2 ppm, MS/MS tolerance = ±0.2 Da, fixed modification = carbamidomethyl (C), variable modification = oxidation (M), charge states = 2+ and 3+, and decoy database on FDR <1%. Quantitative data of each protein was obtained from averaging areas under curve (AUC) (or peak areas) of peptide precursor ion intensity of the three most abundant peptides identified from each protein. Note that background was subtracted from all peak areas.

### Western blotting

Equal amount of total protein (30 µg/lane) from each sample was separated by 12% SDS-PAGE and transferred onto a nitrocellulose membrane. After blocking non-specific bindings with 5% skim milk in PBS for 1 h, the membrane was incubated with mouse monoclonal anti-α-tubulin, anti-vimentin, anti-HSP60, anti-HSP70 or anti-HSP90, or goat polyclonal anti-annexin A2, or rabbit polyclonal anti-α-enolase antibody (all were purchased from Santa Cruz Biotechnology and diluted 1:1,000 in 1% skim milk in PBS) at 4 °C overnight. Note that HSP60 served as the loading control. After probing with corresponding secondary antibody conjugated with horseradish peroxidase at a dilution of 1:2,000 in 1% skim milk in PBS at RT for 1 h, the immunoreactive protein bands were visualized by SuperSignal West Pico chemiluminescence substrate (Pierce Biotechnology, Inc.; Rockford, IL) and autoradiography. Band intensity data was obtained using ImageMaster 2D Platinum version 6.0 (GE Healthcare; Uppsala, Sweden).

### Global protein network analysis

All differentially expressed proteins in lesional vs. non-lesional biopsies from AA patients were subjected to global protein network analysis using Search Tool for the Retrieval of Interacting Genes/Proteins (STRING) version 10.0 (http://string.embl.de). The predicted protein-protein associations were queried through experimentally derived physical protein interactions from literatures combining with the databases of curated biological pathway knowledge^54^. In addition, the annotated gene ontology being enriched according to their biological processes were obtained.

### Statistical analysis

Quantitative proteome data are reported as mean ± SEM of the data obtained from technical triplicates. Comparisons between the two groups of samples were performed using unpaired Student’s *t*-test and Mann-Whitney U test. *P* values less than 0.05 were considered statistically significant.
